# Enhancing Clarity and Patient Safety: Evaluating the Inclusion of Laterality in Orthopaedic Outpatient Correspondence in Galway, Ireland

**DOI:** 10.7759/cureus.60782

**Published:** 2024-05-21

**Authors:** Abobaker Younis

**Affiliations:** 1 Orthopaedics and Traumatology, University Hospital Galway, Galway, IRL

**Keywords:** patient safety, outpatient letters, orthopaedic department, laterality, ireland

## Abstract

Background

In medical documentation, accurate mention of laterality, whether a condition or treatment pertains to the left or the right side of the body, is critical for avoiding errors like wrong-site surgeries, which can have severe repercussions. This study aims to assess compliance regarding the mention of laterality in outpatient clinic letters at Galway University Hospital.

Methods

An analytical cross-sectional study was conducted at Galway University Hospital from February to August. We reviewed outpatient letters in the Orthopaedic Department. The Fisher exact and Chi-square tests were employed to observe the association between the levels of practitioners and laterality.

Results

A total of 278 outpatient letters from 37 practitioners in one week were analyzed. The overall laterality rate was 65.1%. It was observed that there was a male predominance in the workforce (31; 83.8%).

Conclusion

In conclusion, our study revealed satisfactory improvement in the prevalence of mentioning laterality among healthcare providers. It is recommended that educational sessions and re-auditing be carried out to enhance the quality of care.

## Introduction

Accurate medical documentation serves as the backbone of effective healthcare delivery, ensuring that every facet of patient care is based on reliable and precise information [1]. Among the critical details captured in medical records, the mention of laterality - identifying whether a condition or treatment pertains to the left or the right side of the body - holds particular significance. This seemingly minor detail is pivotal in numerous medical specialties, including orthopedics, neurology, and radiology, where the distinction between left and right can influence both diagnostic conclusions and therapeutic interventions [2].

The correct identification of laterality is crucial for patient safety. Errors in laterality can lead to adverse outcomes, such as wrong-site surgeries, one of the most egregious mistakes in medical practice. For instance, performing a surgical procedure on the wrong side of the body not only fails to address the patient's actual medical issue but also subjects them to unnecessary surgical risk and subsequent interventions to correct the error. These incidents, while preventable, continue to occur and can lead to severe physical and psychological distress for patients, tarnish healthcare providers' reputations, and result in significant legal and financial repercussions for healthcare institutions [3,4].

From a legal perspective, laterality errors are often classified under medical malpractice, leading to lawsuits that can financially burden healthcare systems and damage the trust between patients and medical professionals [5]. Ethically, the fidelity to not harm is compromised, shaking the foundational trust that patients place in the healthcare system. Highlighting the importance of laterality in medical records is not only a practical measure but also an ethical imperative to uphold the quality and safety of healthcare services [6].

Accurate documentation, including laterality, enhances the continuity of care by providing subsequent caregivers with clear and precise information, facilitating ongoing patient management. This continuity is vital for chronic conditions and long-term treatments where multiple healthcare providers may be involved over time. Incorrect or incomplete documentation can lead to miscommunication, repeated diagnostics, delayed treatments, and ultimately, compromised patient care outcomes [6].

Following a consultation, the doctor then uses a dictaphone to record the outpatient letter, the letter is subsequently written by the secretariats who transcript. Despite the procedure being performed on a bilateral organ and the side of surgery not always being indicated in the clinic letter. It is to carefully seek that the side is indicated in clinic letters. WHO surgical safety has played an efficient role in minimizing such incidents [7]. The present study was conducted to evaluate compliance by including the laterality in clinic letters besides assessing whether the mentioned side is the correct one.

## Materials and methods

Study design and setting

An analytical cross-sectional study was conducted at Galway University Hospital, a major public health facility serving a diverse population. The study period spanned from February to August 2023, during which time we systematically examined outpatient clinic letters from the orthopedic department.

Inclusion and exclusion criteria

The inclusion criteria were outpatient letters detailing patient interactions for non-bilateral and non-spinal orthopedic conditions. The exclusion criteria were letters involving bilateral injuries, spinal cases, or any elective procedure, as these could potentially skew the importance and representation of laterality.

Data collection

Outpatient letters written by orthopedic practitioners were collected over a continuous period of one week. These letters were collected on a total coverage basis each day to ensure a representative sample of the clinical activities. To maintain a focus on typical cases and enhance the reliability of laterality reporting, letters pertaining to patients with bilateral injuries, spinal cases, and elective surgeries were excluded from the study.

Variables measured

Each letter was reviewed for the clarity of diagnosis documentation. Special attention was given to the mention of laterality (left or right side noted) in the diagnostic descriptions, in addition to whether the mentioned laterality is correct or not.

Data management and analysis

Data extracted from the outpatient letters was entered into Microsoft Excel (Microsoft Corporation, Redmond, WA, US) for initial coding and cleaning. Following this, the data was imported into SPSS (Statistical Package for the Social Sciences) version 23.0 (IBM Corp., Armonk, NY, US) for detailed analysis. Descriptive statistics, such as means, and standard deviations were computed for quantitative variables. Categorical variables were analyzed using frequencies and percentages. To assess the association between the level of the practitioner (Senior House Officer, Registrar, Consultant) and the accuracy of laterality documentation, Fischer's exact and Chi-square tests were utilized. A p-value of less than 0.05 was considered statistically significant, indicating a meaningful difference or association.

Intervention

To enhance the accuracy of laterality documentation in outpatient clinic letters, a multifaceted intervention was implemented. This included educational sessions for healthcare providers focusing on the importance of specifying laterality in medical records. Additionally, reminder stickers were strategically placed within the clinic as visual prompts to ensure laterality mention. We also liaised with secretarial staff responsible for transcribing doctors' notes, reinforcing the need to accurately transcribe diagnostic information, including laterality. Following these interventions, a reaudit was conducted, analyzing the same number of patients over a week to assess any improvements in the documentation practices.

Ethical considerations

In conducting this study, strict confidentiality was maintained to protect the identities of both patients and doctors. All personal and clinical information was anonymized before analysis to ensure privacy. The Institutional Review Board (IRB) deemed formal approval not applicable, as the study did not involve direct intervention on patients. Instead, the focus was on improving documentation practices within existing clinical processes, ensuring adherence to ethical standards in observational research.

## Results

Our analysis encompassed 278 outpatient letters drafted by 30 practitioners over one week at Galway University Hospital. The demographic breakdown, as depicted in Table 1, revealed a notable male predominance among the practitioners who contributed to the outpatient letters, with males accounting for 82.4% of the sample. This demographic distribution aligns with broader trends in the medical field, particularly in orthopedics, where male practitioners predominate.

**Table 1:** Demographics and clinician grades

Variables	N	%
Gender		
Male	28	82.4
Female	6	17.6
Level		
Senior House Officer (SHO)	12	35.2
Registrar	11	32.4
Consultants	11	32.4

Laterality mention rates

**Figure 1:**
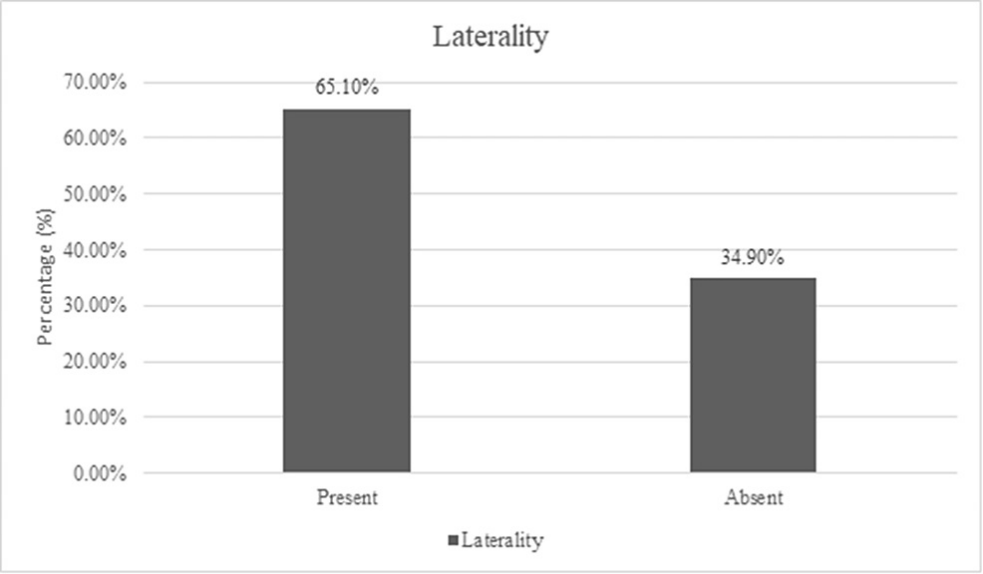
Overall percentage of outpatient letters documenting laterality during the study period This graph highlights the initial rate of laterality mentions before the intervention.

**Figure 2:**
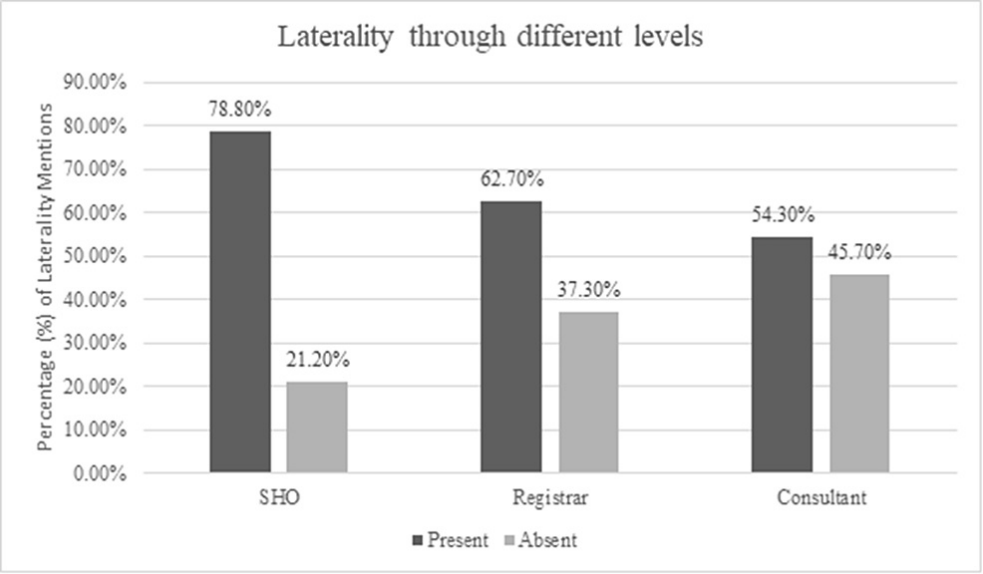
Percentage of including laterality among the level of practitioners

In our study, the overall rate of laterality mentioned in outpatient letters was 65.1%, as shown in Figure 1. This rate reflects significant room for improvement given the critical role of laterality in avoiding surgical errors. Figure 2 illustrates the laterality among the level of practitioners; SHO, registrar, and consultant.

**Figure 3:**
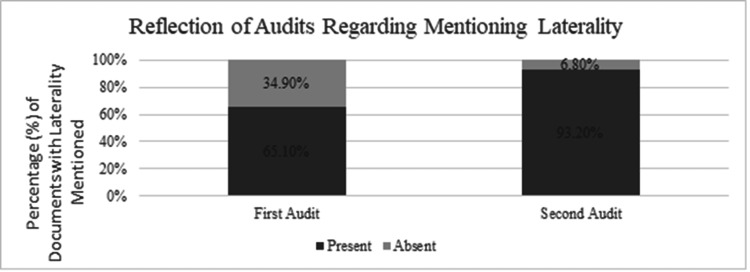
Comparing the rates of laterality mentions in outpatient letters before and after the intervention across two audit periods

The two audits conducted during the study period further highlight trends in documentation practices: initially, laterality was mentioned in 65.1% of cases when it was applicable. However, by the second audit, this rate had substantially increased to 93.2%, demonstrating a marked improvement in compliance following targeted interventions at our institution (Figure 3).

Association between practitioner level and laterality mention

Table 2 presents the association between the level of practitioners and the frequency of laterality mention, which was found to be statistically significant (P<0.05). Senior House Officers (SHO) had the highest laterality mention rate at 78.8% while consultants had the lowest at 54.3%. This variation suggests that less experienced practitioners may adhere more strictly to protocol or may benefit more from recent training on documentation practices.

**Table 2:** Statistical analysis results detailing the association between the level of practitioners and the inclusion of laterality in outpatient letters

Variables	Literality		P-Value
Level of practitioners	Present	Absent	
Registrar	62.7%	37.3%	<0.05
SHO	78.8%	21.2%	
Consultant	54.3%	45.7%	

Reflecting on audits and compliance improvement

Figure 3 illustrates the outcomes of our focused audits on laterality mentions. The substantial increase from 65.1% to 93.2% in laterality mentioned by the second audit underscores the effectiveness of the interventions implemented such as enhanced training sessions and reminders within the outpatient departments. These findings highlight the potential for significant improvements in documentation accuracy through relatively simple administrative measures.

## Discussion

The results from this study illuminate several key aspects of medical documentation in an orthopedic trauma clinic setting, where patients are treated either nonoperatively or postoperatively. First, the significant discrepancy in laterality mentions among different levels of practitioners underscores the need for a unified approach to training and compliance monitoring [8]. This is particularly vital in a trauma clinic, where accurate documentation of laterality can guide decision-making processes about potentially necessary surgeries following an initial nonoperative approach [9]. While the immediate risk of wrong-site surgery is lower in nonoperative care, the eventual decision to operate, if taken, depends critically on precise and reliable medical records [10]. Second, the marked improvement in laterality mentions between the first and second audits highlights the effectiveness of ongoing feedback and training [11]. These interventions are crucial, especially considering that for some patients, the choice to undergo surgery may arise after a period of nonoperative treatment. Through diligent efforts to enhance the clarity and accuracy of medical records, especially concerning laterality, we can significantly reduce the risk of adverse events, thereby improving patient outcomes and maintaining high standards of care.

Errors in laterality assessment are among the most severe and detrimental mistakes in the field of medicine. The presence of bilateral representation in a body part poses a potential risk of performing surgical or procedural interventions on the incorrect side of the body. Only a small number of surgical specialties are exempt from the potential risk of wrong-sided events taking place [12].

Efforts made by various organizations have resulted in a reduction of occurrences of 'never events'; however, these events persist [11,13]. According to a study, there are numerous cases where the side is not described completely, though incorrect side operation was not carried out. This practice of leaving out the side could be the beginning of a series of events that lead to a catastrophic incident [7].

In 2016, a total of 179 instances of incorrect-site surgeries were reported to the Strategic Health Authorities in England, with a significant number of these cases involving errors related to right-left (RL) disparity surgical specialties are not the sole clinical disciplines in which RL errors may arise. Additional documented occurrences encompass ocular injections, neural blockade, radiation therapy, and thoracentesis (NHS improvement). The overall laterality rate was higher than 65%. In another study, the prevalence of missing laterality was found to be 8.71% [7].

In a research study in the neurology department [14], it was observed that Senior House Officers (SHO) exhibited the highest level of compliance. However, when comparing compliance rates across different categories of clinicians, the differences were found to be statistically insignificant. These findings are consistent with our results; however, we observed a statistically significant association. Concerning Stroke Consultants, specifically Consultant Physicians/Internists, only a minority of 3.1% demonstrated adherence to the practice of inquiring about the laterality of patients. This aspect holds significant importance in the context of stroke patients.

This study is subject to several limitations that should be considered when interpreting the findings. First, the duration of the study was confined to one week, which may not adequately capture variations in patient outcomes, treatment effectiveness, or hospital practices that could occur over a longer period. This limited timeframe may restrict the generalizability of the results to other periods with potentially different clinical workflows or patient populations. Second, the study was conducted in a single hospital setting. While this provides detailed insights specific to this environment, the findings may not apply to other hospitals with different patient demographics, resources, or organizational structures. The unique characteristics of the hospital, such as staff expertise, available technology, and patient management protocols, could influence the study outcomes and may not represent typical conditions in other settings.

## Conclusions

Our study conducted at Galway University Hospital has provided valuable insights into the practice of documenting laterality in orthopedic outpatient letters. We observed a marked improvement in the mention of laterality - from 65.1% to 93.2% - following the implementation of targeted educational interventions and periodic audits. This enhancement not only signifies a direct benefit to patient safety by potentially reducing the risk of wrong-site surgeries but also underscores the effectiveness of simple, systematic changes in healthcare practices. Despite these positive outcomes, the study highlighted the need for ongoing education and standardized practices across all levels of medical professionals, from Senior House Officers to consultants, to maintain high standards of clinical documentation. The significant discrepancies in compliance rates among different levels of practitioners indicate an opportunity for tailored training programs that address specific needs and challenges within the hospital setting.
